# Fasciolosis: Analytical approaches to post-treatment liver enzyme activity changes in cattle

**DOI:** 10.2478/helm-2026-0006

**Published:** 2026-06-15

**Authors:** Ľ. BURCÁKOVÁ, A. KÖNIGOVÁ, M. BABJÁK, Z. KOSTECKÁ, M. VÁRADY1

**Affiliations:** 1 Institute of Parasitology, Slovak Academy of Sciences, Hlinkova 3, 040 01 Košice, Slovakia; 2 Department of Chemistry, Biochemistry and Biophysics, University of Veterinary Medicine and Pharmacy, Komenského 73, 040 01 Košice, Slovakia

**Keywords:** *Fasciola hepatica*, liver flukes, post-treatment enzyme changes, gamma-glutamyl transferase, biochemical parameters, chronic fasciolosis

## Abstract

The study investigated the applicability of three different analytical approaches for assessing post-treatment changes in liver enzyme activity following anthelmintic therapy over a 21-day period in cattle naturally infected with fasciolosis. A total of 48 animals were allocated into four groups of 12 cows each: one untreated control group and three treatment groups. Treated cattle received the following anthelmintic combinations: ivermectin plus clorsulon (IVM+CLOR), levamisole plus oxyclozanide (LEV+OXY), and albendazole (ABZ). Biochemical assessments were performed using standardized kinetic and colorimetric methods in accordance with the International Federation of Clinical Chemistry and Laboratory Medicine (IFCC) guidelines. Pre- and post-treatment changes in hepatic enzymes (aspartate aminotransferase – AST, alanine aminotransferase – ALT) and cholestatic enzymes (alkaline phosphatase – ALP, gamma-glutamyl transferase – GGT) were evaluated. The analyses demonstrated comparable applicability and reliability of quantifying post-treatment enzyme changes when referenced either to pre-treatment or to the control group at the group level, rather than at the individual level. Among the enzymes assessed, all three analytical approaches showed agreeing results of significantly decreased GGT activity by 3.8 – 32.4 % and 21.6 – 47.5 % across treatment groups on Day 7 and 21, respectively, following flukicide administration. These findings underscore the importance of quantifying liver enzyme changes and highlight the relevance of GGT post-treatment alterations. The observed associations between flukicide therapy and biochemical liver profiles may provide a foundation for future studies aimed at elucidating the impact of treatment on clinic-biochemical patterns and the potential for liver recovery following the resolution of chronic fasciolosis in naturally infected cattle.

## Introduction

Fasciolosis is widely recognized as an economically relevant disease in ruminants, primarily due to liver condemnation resulting from irreversible tissue damage caused by migrating flukes throughout the hepatic parenchyma (Bosco *et al.*, 2023; Hayward *et al*., 2021; Mehmood *et al.*, 2017). Accurate detection of ongoing *Fasciola hepatica* infection remains challenging because current diagnostic approaches rely on detecting *Fasciola* -like eggs in feces. However, a positive coprological examination does not necessarily reflect the clinical course of the disease, since egg shedding occurs predominantly during the later stages of infection (Dorchies, 2007; Corrales *et al.*, 2023).

Given these limitations, non-specific diagnostic tools, such as serum liver biochemistry tests, may be helpful in assessing fasciolosis (Katsoulos *et al.,* 2009; Zaitsev *et al.*, 2020). Elevated liver enzyme activities may indicate impaired hepatic synthetic capacity, hepatocellular injury, or inflammatory processes such as cholangiohepatitis (Coppo *et al.*, 2011; Kowalczyk *et al.,* 2018; Neira *et al.*, 2024), which are clinico-pathological findings commonly observed in animals infected with *F. hepatica* trematodes (Corrales *et al.*, 2023). In contrast, a post-treatment decline in liver enzyme activity may suggest effective therapy and/or hepatic recovery following successful fluke elimination (Benchaoui & McKellar, 1993; Costa *et al.*, 2022; Kozat & Denishan, 2010).

This phenomenon has been investigated in naturally acquired liver fluke infections by Königová *et al.* (2024) and Gedefaw *et al.* (2025), who reported changes in enzyme activity indicative of improved liver function following specific anti-trematode treatments. Manga-González *et al.* (2004) quantified the dynamics of biochemical parameters in experimental ovine dicrocoeliosis, while Königová *et al.* (2024) applied a study-design-adjusted formula to quantify post-treatment biochemical alterations in naturally acquired disease. Overall, data on bovine fasciolosis remain limited, and to our knowledge, no study has yet reported the analytical approaches for estimating post-treatment liver enzyme changes in cattle with fasciolosis.

Therefore, this study aimed to investigate, calculate, and compare three different analytical approaches for evaluating post-treatment changes in hepatic (aspartate aminotransferase – AST, alanine aminotransferase – ALT) and cholestatic (gamma-glutamyl transferase – GGT, alkaline phosphatase – ALP) enzymes in cattle naturally infected with fasciolosis.

## Material and Methods

### Study design

The experimental herd consisted of 48 Charolaise cattle, including 39 females and 9 males at the age of 3-7 years of reproductive age with the mean weight of 571.5 kg. Based on positive *F. hepatica* coprological testing determining mean baseline faecal egg counts of 16.9-73.3, four experimental groups were established: three treatment groups (IVM+CLOR – ivermectin + clorsulon; LEV+OXY – levamisole + oxyclozanide; ABZ – albendazole) and one untreated control group (Table 1). Each group equally consisted of 12 animals. Blood samples were collected on three sampling days: Day 0 (pre-treatment), Day 7, and Day 21 (post-treatment), to evaluate changes in liver enzyme activity following deworming. The assessed enzymes included alanine aminotransferase (ALT), aspartate aminotransferase (AST), gamma-glutamyl transferase (GGT), and alkaline phosphatase (ALP). Quantification of enzyme activity changes was performed by comparing enzyme activities in treated animals on Day 7 and 21 either with those of untreated control group (Formulas 1 and 2) or with the untreated animals sampled on Day 0 (Formula 3). The experimental herd had not received any anthelmintic treatment for at least 12 months prior to the start of the study, and access to pasture was fully restricted for the duration of the experiment.

**Table 1.** Anthelmintic schedule for control and treatment groups.

Group	Control	IVM+CLOR*	LEV+OXY*	ABZ*
Trade name	-	Ivomec Super Injection® (MERIAL SAS, France)	Interzan Gold Oral® (Interchemie, Netherlands)	Albendavet 10%^®^ (DIVASA-FARMAVIC S.A., Spain)
Effective ingredient	-	ivermectin, clorsulon	levamisole, oxyclozanide	albendazole
Dosing	-	1 ml per 50 kg b.wt., parenteral	2.5 ml per 10 kg b.wt., peroral	1 ml per 10 kg b.wt., peroral

1 *- treatment groups

### Biochemical analyses

Following collection, blood samples were maintained at approximately 4 °C under cool, dry conditions during transport. Serum was obtained by centrifugation at 3000 × g for 10 minutes and separated from the blood clot. Changes in absorbance (ΔA/min) for liver enzymes were determined spectrophotometrically (visible spectrophotometer, ONDA V-10 PLUS, Labbox, Spain) using BIO-LA-TEST® kits (Erba Lachema Ltd., Czech Republic) and an Aqualine AL12 water bath (Lauda, Germany), in accordance with the guidelines of the International Federation of Clinical Chemistry and Laboratory Medicine (IFCC) (Schumann *et al.,* 2010, 2011). Alanine aminotransferase (ALT) and aspartate aminotransferase (AST) activities were determined using a colorimetric method according to Reitman & Frankel (1957), with minor modifications regarding reagent volumes and incubation times. The catalytic activities of gamma-glutamyl transferase (GGT) and alkaline phosphatase (ALP) were assessed using a kinetic colorimetric method (Persijn & van der Slik, 1976), with enzyme-specific adjustments. Enzyme activities were expressed as international units per litre (IU/L). Percentage enzyme activity change (%EC) was calculated as a percentage of decrease or increase at both individual and group levels using the following analytical approaches:

Formula 1:

$$\begin{array}{l} \%\text{EC}1=(\;\text{control}\;\text{EA}\;-\text{post-treatment}\;\text{EA})\times 100/\;\text{post-treatment}\;\text{EA}\;\\ \left(\;\text{Manga-Gonz}\text{á}\text{lez}\;et\;al\text{.,}\;2004\right) \end{array}\;$$

Formula 2:

$$\begin{array}{l} \text{\%EC2 = [(control EA }-\text{ post-treatment EA) / control}\;\text{EA] × 100}\\ \text{(Coles}\;et\;al\text{.,}\;\;\text{1992)} \end{array}$$

Formula 3:

$$\begin{array}{l} \text{\%EC3 = [1}-\text{(after treatment EA/ before treatment EA)] × 100}\\ \text{(Kochapakdee }et\;al\text{.,1995)} \end{array}$$

### Data analyses

Differences in enzyme activity changes across treatment days and groups were evaluated using the Kruskal-Wallis test, with Bonferroni correction applied to p-values. Pairwise comparisons were subsequently conducted using the Mann-Whitney U test in SPSS® statistical software. A significance level of p < 0.05 was applied for all statistical analyses.

## Ethical Approval and/or Informed Consent

All procedures performed in this study were in accordance with the ethical standards of the Ethics Committee on 17-November-2021 and met the requirements of the Ethics Committee of the Institute of Parasitology of the Slovak Academy of Sciences, in accordance with the national legislation in Slovakia – Animal Welfare Act No. 23/2009, and were approved on 1-January-2022.

## Results

### Individual enzyme activity change

Analysis of individual enzyme activity changes revealed heterogeneous post-treatment responses, with enzyme-specific tendencies toward increased or decreased activity (Table 2). For aspartate aminotransferase (AST), the percentage of activity increases and decreases differed significantly (Mann-Whitney U = 8937, p < 0.001). Specifically, the results of Formulas 1 and 2 calculations were associated with a predominant decrease in AST activity, whereas Formula 3 resulted in a consistent increase in AST activity.

**Table 2.** The average individual calculations of the enzyme activity change using Formula 1 (F1), 2 (F2) and 3 (F3) showing different numbers of animals (n) with increased and decreased activity on Day 7 and 21.

Enzyme	Day	Increased enzyme activity (%)						Decreased enzyme activity (%)					
		F1	*n* ^1^	F2	*n* ^2^	F3	*n* ^3^	F1	*n* ^1^	F2	*n* ^2^	F3	*n* ^3^
AST	7	11.6	8	15.0	8	61.0	34	16.7	28	13.4	28	23.2	2
	21	4.4	4	4.7	4	72.5	36	13.4	32	11.3	32	0	0
ALT	7	28.3	21	50.0	21	62.5	23	66.2	15	33.2	15	28.2	13
	21	17.7	9	25.3	9	0	0	62.9	27	33.1	27	36.4	36
ALP	7	50.0	17	>100	17	55.0	24	>100	19	50.1	19	35.8	12
	21	46.8	15	>100	15	39.3	27	>100	21	51.3	21	32.8	9
GGT	7	13.4	10	17.7	10	15.8	10	47.8	26	28.3	26	32.9	26
	21	12.2	8	14.3	8	24.7	5	54.0	28	31.0	28	31.6	31

Similarly, a significant difference between post-treatment increases and decreases was observed for GGT activity (Mann-Whitney U = 1622, p < 0.001), indicating a higher proportion of activity reductions following treatment. In contrast, alanine aminotransferase (ALT) (Mann-Whitney U = 4753, p = 0.723) and alkaline phosphatase (ALP) (Mann-Whitney U = 4208, p = 0.500) exhibited comparable outcomes of increased and decreased activity, with no statistically significant differences detected.

Although Formulas 1 and 2 were derived from comparisons with the control group, the calculated percentages of enzyme activity changes differed significantly between these two formulas for decreased ALT activity (p < 0.05), for both increased (p < 0.05) and decreased (p < 0.01) ALP activity, and for decreased GGT activity (p < 0.01). In contrast, aspartate aminotransferase (AST) activity yielded comparable results for both calculations. Comparisons involving Formula 3 with Formulas 1 and 2 demonstrated more pronounced differences (Table 3).

**Table 3.** Comparisons between the calculations of enzyme activity changes using Formula 1,2 and 3 analyzed by Kruskal-Wallis test: NS – not significant, level of significance at p < 0.05.

		AST	ALT	ALP	GGT
increase	Formula 1 / Formula 2	NS	NS	p<0.05	NS
	Formula 1 / Formula 3	p<0.001	p<0.001	NS	NS
	Formula 2 / Formula 3	p<0.001	p<0.05	p<0.001	NS
decrease	Formula 1 / Formula 2	NS	p<0.05	p<0.01	p<0.01
	Formula 1 / Formula 3	NS	p<0.05	p<0.001	p<0.05
	Formula 2 / Formula 3	NS	NS	NS	NS

### Group enzyme activity change

The temporal dynamics of enzyme activity across treatment groups, calculated using Formulas 1-3, are summarized in Table 4. Aspartate aminotransferase (AST) activity increased over the study period in all treatment groups. Statistically significant increases (46.0 – 85.5 ^¢^%) were observed only in comparisons with Day 0, indicating longitudinal changes within the same animals. In contrast, comparisons with the control group revealed minimal and largely non-significant differences (3.1 – 12.2 ^¢^%). AST activity was consistently higher on Day 21 than on Day 7 across all treatments; however, only the IVM+CLOR group showed a significant increase between the two time points. No significant differences were observed between treatment groups, suggesting that AST activity was not substantially influenced by treatment choice.

**Table 4.** Calculations of percentages of after treatment enzymes change with level of significance analyzed by Kruskal-Wallis test: ^NS^ – not significant, * – p < 0.05; ** – p < 0.01; *** – p < 0.001.

Enzyme	Group	Day	Formula 1 (%)	EC	Formula 2 (%)	EC	Formula 3 (%)	EC
	ABZ	7	9.7^NS^	I	10.7^NS^	I	47.8^***^	I
		21	10.5^NS^	I	11.7^NS^	I	61.1^***^	I
AST	LEV+	7	10.9^*^	I	12.2^*^	I	46.0^***^	I
	OXY	21	10.9^*^	I	12.2^*^	I	57.9^***^	I
	IVM+	7	3.1^NS^	I	3.2 ^NS^	I	58.6^**^	I
	CLOR	21	10.5 ^NS^	I	11.7 ^NS^	I	85.5^***^	I
	ABZ	7	24.1^***^	D	19.5^***^	D	10.8 ^NS^	I
		21	60.7^***^	D	37.8^***^	D	34.6 ^NS^	D
ALT	LEV+	7	27.3^***^	D	21.5^***^	D	31.3 ^NS^	D
	OXY	21	9.1^***^	D	8.3^***^	D	38.7^*^	D
	IVM+	7	37.6 ^NS^	I	60.4 ^NS^	I	86.7^*^	I
	CLOR	21	67.2^***^	D	40.2^***^	D	46.8^***^	D
	ABZ	7	28.4 ^NS^	I	39.6 ^NS^	I	24.1 ^NS^	I
		21	28.6 ^NS^	I	40.0 ^NS^	I	55.6 ^NS^	I
ALP	LEV+	7	18.6 ^NS^	I	22.9 ^NS^	I	13.5 ^NS^	I
	OXY	21	1.7 ^NS^	D	1.7 ^NS^	D	13.5 ^NS^	I
	IVM+	7	11.1 ^NS^	I	12.5 ^NS^	I	25.6 ^NS^	I
	CLOR	21	21.9 ^NS^	D	17.9 ^NS^	D	14.5 ^NS^	I
	ABZ	7	27.1^***^	D	21.3^***^	D	19.8^*^	D
		21	47.5^***^	D	32.2^***^	D	21.6^**^	D
GGT	LEV+	7	13.0^***^	D	11.5^***^	D	32.5^*^	D
	OXY	21	34.1^***^	D	25.4^***^	D	35.3^**^	D
	IVM+	7	4.0^***^	D	3.8^***^	D	7.4 ^NS^	D
	CLOR	21	31.1^***^	D	23.7^***^	D	16.7 ^NS^	D

1 EC – enzyme change, I – increased enzyme activity, D – decreased enzyme activity.

Alanine aminotransferase (ALT) activity generally decreased (8.3 – 86.7 ^¢^%) across all groups and sampling days, except in the IVM+CLOR group on Day 7 and in the ABZ group, where increases were observed. On Day 7, the significance of ALT changes varied between groups. By Day 21, all decreases in ALT activity (8.3 – 67.2 ^¢^%) were statistically significant in the LEV+OXY and IVM+CLOR groups, irrespective of the formula applied. Comparisons between Day 21 and Day 7 showed that, in the LEV+OXY group, Formulas 1 and 2 indicated decreased activity, whereas Formula 3 suggested an opposite trend; none of these changes were significant. In the IVM+CLOR group, significant changes were detected on Day 21 (p < 0.001), although the direction of change differed among formulas. In the ABZ group, despite non-significant changes relative to baseline, ALT activity increased significantly from Day 7 to Day 21 (p < 0.01). Between-treatment comparisons demonstrated significant differences on Day 7 between the IVM+-CLOR group and both the ABZ and LEV+OXY groups, and on Day 21 between IVM+CLOR and LEV+OXY, with IVM+CLOR inducing the most pronounced ALT alterations.

A significant reduction in alkaline phosphatase (ALP) activity was observed in the ABZ group on Day 7 (19.8 – 27.1 %), with a greater decline on Day 21 (21.6 – 47.5 %), independent of the formula applied. However, these changes were not significant when comparing Day 7 with Day 21, nor were they influenced by treatment selection.

Gamma-glutamyl transferase (GGT) activity showed a consistent decreasing trend across all treatments, time points, and formulas. Non-significant changes were observed only in the IVM+CLOR group when assessed using Formula 3. Overall, GGT activity decreased significantly by 4.0 – 35.3 % on Days 7 and 21 relative to Day 0. Although reductions were more pronounced on Day 21, the difference between Days 7 and 21 was not significant. No significant differences were detected between treatment groups, indicating comparable GGT responses across anthelmintic regimens. Comparisons of enzyme activity changes calculated using Formulas 1, 2, and 3 did not reveal statistically significant differences, yielding comparable results regardless of the reference group used, whether an independent untreated control group or the same animals assessed longitudinally over time.

## Discussion

In the present study, the applied analytical approaches revealed significantly increased AST activity, variable (decreasing-increasing) trends in ALT activity, and decreased GGT activity across treatment groups across the study period, both at the individual and group levels. Upon individual-level comparisons of enzyme activity changes, the direction of change (increase or decrease) showed considerable differences when animals were accounted as individual units. However, individual trends indicating increased AST activity and decreased ALT and GGT activities were generally consistent with the group level outcomes. Therefore, tendencies toward increases or decreases in liver enzyme activity could be reliably evaluated at the group level.

Although different outcomes were obtained when applying the for-mulas to individual comparisons, group level enzyme activity changes did not yield discrepant results. Formulas 1 (Manga-González *et al.*, 2004) and 2 (Coles *et al.*, 1992) were applied to evaluate post-treatment differences relative to the untreated control group. The latter approach is routinely applied to determine treatment efficacy in the unpaired design of the *in vivo* faecal egg count reduction test (FECRT) for the assessment of anthelmintic treatment efficacy, as standardised by the World Association for the Advancement of Veterinary Parasitology (WAAVP). In addition, an alternative calculation proposed by Kochapakdee *et al.* (1995) (Formula 3) was applied in our study to quantify post-treatment changes in enzyme activity, based on time-dependent comparisons with untreated animals on Day 0. This paired-design approach is also recommended by WAAVP for FECRT treatment efficacy evaluation. At the group level, all applied analytical approaches yielded comparable results, indicating that the estimation of liver enzyme activity changes following anthelmintic treatment was independent of the formula used. Collectively, these findings suggest that post-treatment enzyme activity changes are not strictly dependent on the reference group used for comparison, whether assessed within the same animals relative to pre-treatment or relative to an additional untreated control group. The WAAVP guidelines recommend using both paired and unpaired designs in animals infected with *Fasciola hepatica* to improve objectivity when evaluating reductions in egg counts (Burden *et al.,* 2014; Dobson *et al.*, 2012). Based on this principle, a similar combined analytical approach may apply to liver biochemical profiling, where using both calculations could enhance the interpretability of results.

To our knowledge, studies quantifying changes in liver enzyme activity following anti-*Fasciola* treatment are scarce. Regarding aminotransferases, the most consistent findings were observed for AST, which showed a clear increase on Days 7 and 21. Although ALT is considered a more sensitive marker of hepatocellular dysfunction (Pratt & Kaplan, 2000), our results showed inconsistent patterns of increasing and decreasing across treatment days, yielding divergent outcomes depending on the analytical approach used. The AST is a non-liver-specific enzyme, and its elevation may reflect injury to other organ systems, including the cardiovascular, skeletal, urogenital, or neurological tissues. Therefore, given the limited liver specificity of AST, its activity should be interpreted in conjunction with ALT. Thus, increased AST activity without abnormal increases in ALT activity could be influenced by AST’s lower hepatic specificity, as well as by a variety of factors of non-hepatic origin. Nevertheless, aminotransferases predominantly reflect acute hepatic damage caused by migrating liver flukes rather than chronic stages of infection (Center, 2007; Costa *et al.*, 2022; Kaneko *et al.*, 2008; Wang *et al.*, 2019). Moreover, the animals’ age (3 – 7 years) and semi-intensive management system may have led to prolonged, cumulative infections, ultimately resulting in a chronic, subclinical course of infection. These observations support the presumption that chronic fasciolosis was present in our study cohort.

Among cholestatic enzymes, significant alterations in GGT activity were observed in the present study and were consistently confirmed by both analytical approaches. According to Formulas 1/2, GGT activity decreased significantly by 3.8 – 47.5 % across treatment groups and days. Formula 3 revealed a decrease by 19.8 – 35.3 % in LEV+OXY and ABZ groups, whereas IVM+CLOR treatment did not significantly influence the GGT activity decrease. These results could potentially suggest an improvement in liver function following antitrematodal therapy. Our findings are consistent with previous reports documenting reductions in GGT activity following anthelmintic treatment in naturally infected ruminants (Elitok *et al.*, 2006; Gedefaw *et al.*, 2025; Ibrahiem *et al.*, 2023; Shrimali *et al.*, 2016). Similarly, Königová *et al.* (2024) reported decreased GGT activity in response to ABZ treatment against *Dicrocoelium dendriticum*, further supporting the association between liver-related trematodes and alterations in GGT activity. Moreover, elevated GGT activity has been widely reported in comparisons between infected and non-infected ruminants with naturally acquired fasciolosis (Coppo *et al.*, 2011; El-Aziem & Mohamed, 2017; Hodžić *et al.*, 2013; Matanović *et al.*, 2007; Mooney *et al.*, 2009; Neira *et al.*, 2024), as well as in chronic experimental infections (Costa *et al.*, 2022; Hutchinson *et al*., 2009; Phiri *et al*., 2007; Raadsma *et al.*, 2007; Yang *et al.*, 1998). Therefore, a post-treatment decline in GGT activity may reflect functional improvement and reparative processes within the hepatic parenchyma following elimination of liver flukes.

In conclusion, the present findings highlight the importance of quantitatively assessing post-treatment changes in liver enzyme activity. The consistent decrease in GGT activity across all treatment groups may indicate a direct association between antitrematodal therapy and favourable biochemical changes in the liver, supporting its potential role in indicating a hepatic recovery in chronic bovine fasciolosis.
